# Augmenting Cognitive Function in the Elderly with Mild Cognitive Impairment Using Probiotic *Lacticaseibacillus rhamnosus* CBT-LR5: A 12-Week Randomized, Double-Blind, Parallel-Group Non-Comparative Study

**DOI:** 10.3390/nu17040691

**Published:** 2025-02-14

**Authors:** Su-Jin Jung, Kyohee Cho, Eun-Soo Jung, Dooheon Son, Jong-Seon Byun, Song-In Kim, Soo-Wan Chae, Jong-Chul Yang, Seung-Ok Lee, Sanghyun Lim

**Affiliations:** 1 Clinical Trial Center for Functional Foods, Jeonbuk National University Hospital, Jeonju 54907, Republic of Korea; sjjeong@jbctc.org (S.-J.J.); esjung@jbctc.org (E.-S.J.); swchae@jbctc.org (S.-W.C.); yangjc@jbnu.ac.kr (J.-C.Y.); 2 Clinical Trial Center for K-FOOD Microbiome, Biomedical Research Institute, Jeonbuk National University Hospital, Jeonju 54907, Republic of Korea; 3 R&D Center, Cell Biotech Co., Ltd., Gimpo-si 10003, Republic of Korea; khcho@cellbiotech.com (K.C.); dhson@cellbiotech.com (D.S.); jsbyen@cellbiotech.com (J.-S.B.); sikim@cellbiotech.com (S.-I.K.); 4 Department of Psychiatry, Jeonbuk National University Medical School, Jeonju 54896, Republic of Korea; 5 Department of Gastroenterology and Hepatology, Jeonbuk National University Hospital, Jeonju 54896, Republic of Korea; 6 Department of Internal Medicine, Jeonbuk National University Medical School, Jeonju 54896, Republic of Korea

**Keywords:** mild cognitive impairment (MCI), *Lacticaseibacillus rhamnosus* CBT-LR5, skim milk, probiotics, cognitive function, clinical trial

## Abstract

Background: Probiotics have been shown to enhance cognitive function in individuals with mild cognitive impairment (MCI), but their efficacy varies, depending on the strain and dosage. Objectives: Clinical investigations are crucial to confirm their safety, efficacy, and mechanism of action. This study was designed to assess the effective dosage, safety, and efficacy of MH-Pro, a test product containing *Lacticaseibacillus rhamnosus* CBT-LR5 (LR5) and skim milk (non-fat dry milk), in improving cognitive function and related physiological changes in older adults suspected of MCI over 12 weeks. Methods: In total, 20 participants (mean age: 68.9 years) were randomly assigned in a 1:1 ratio to either a low-dose group (1 × 10^10^ CFU LR5 with 1622 mg) or a high-dose group (1 × 10^10^ CFU LR5 with 4055 mg skim milk) in a double-blind, parallel-group clinical trial. Results: After 12 weeks, the low-dose group showed significant improvements in the MOCA-K subdomains, specifically in naming (*p* = 0.01) and delayed recall (*p* = 0.003). Additionally, levels of amyloid-β1 40/42 in the blood significantly decreased (*p* = 0.03) following supplementation in the low-dose group. The high-dose group exhibited significant improvement in orientation (*p* = 0.05). Moreover, overall cognitive enhancement was observed in the low-dose group (*p* = 0.003), while the high-dose group showed a trend toward improvement (*p* = 0.06). Fecal analysis revealed significant changes in bacterial composition, with an increase in *Lacticaseibacillus* after 12 weeks of MH-Pro consumption. Together, these findings provide foundational evidence suggesting that MH-Pro supplementation may serve as a potential intervention for enhancing cognitive function through gut–brain axis pathways in the elderly population. However, given the small sample size and the predominance of female participants, the impact of the outcome may be limited. Further large-scale studies are necessary to validate these preliminary results. Conclusions: This study provides foundational evidence to recognize the use of LR5 and skim milk to prepare a probiotic supplement that enhances cognitive function in the aging population.

## 1. Introduction

The global aging population is being driven by enhanced healthcare, decreasing birth rates, and demographic transitions, resulting in a more significant proportion of elderly individuals within society. Specifically, the proportion of the elderly population is expected to rise to 22% by 2050, which is nearly double the current rate [1]. This demographic shift will heighten concerns about the diseases prevalent among the elderly population. The 2023 Dementia Status Report from the Korean Central Dementia Center (KDRC) reported that around 940,000 individuals aged 65 and older were diagnosed with dementia in 2022. The figure is projected to increase to 1.05 million by 2024, 2.26 million by 2040, and 3.34 million by 2070. Additionally, the medical costs associated with dementia were estimated to be KRW 2.8 trillion in 2022, a 58.6% increase from KRW 1.6 trillion in 2015 [2]. Similarly, the Alzheimer’s Association, USA reported that about 7 million elderly individuals in the United States are currently affected by dementia, and the associated caregiving costs are projected to reach USD ~360 billion by 2024 [3]. These data highlight that the financial burden, coupled with dementia-related concerns in older people, substantially affects quality of life. Thus, there is an urgent need for effective strategies to improve the health of the elderly and minimize healthcare expenditures.

Mild cognitive impairment (MCI) is an intermediate stage between normal aging-related cognitive decline and more serious conditions like dementia. Individuals with MCI experience noticeable cognitive changes, such as memory loss or difficulty with thinking skills, but these changes are not severe enough to interfere significantly with daily life. Some people with MCI remain stable, while others may progress to dementia, including Alzheimer’s disease (AD). Dementia is a broad term that refers to a decline in cognitive abilities severe enough to interfere with daily activities. It is not a single disease but a syndrome caused by various brain disorders. Dementia affects memory, reasoning, problem-solving, language, and even personality. Common types of dementia include AD, vascular dementia, frontotemporal dementia, Lewy body dementia, etc. AD is the most common cause of dementia, accounting for 60–80% of cases. It is a progressive neurodegenerative disorder characterized by the accumulation of amyloid-beta plaques and tau tangles in the brain. Symptoms include memory loss, confusion, language difficulties, and impaired problem-solving. AD worsens over time, eventually leading to severe cognitive and functional impairment [4]. Currently, FDA-approved acetylcholinesterase inhibitors, such as donepezil, rivastigmine, lecanemab, donanemab and memantine, and N-methyl-D-aspartate (NMDA) receptor antagonist, are prescribed to treat dementia [5]. However, these medications offer temporary cognitive improvements, and several side effects have been reported [6,7]. Thus, investigation into non-pharmacological treatments, such as the Mediterranean diet and multidomain lifestyle interventions, has been consistently explored to reduce the side effects and enhance the effects of these drugs [8,9]. Also, these non-pharmacological treatments potentially help improve cognitive function and overall health while avoiding the side effects of medication.

Probiotics have long been recognized for their benefits beyond bowel function, including beneficial effects on Type 2 diabetes, antioxidant activity, anti-inflammatory responses, and allergies [10,11,12,13]. Interestingly, recent investigations confirmed the impact of gut microbiota imbalances on the brain, indicating that such imbalances may lead to neurodegenerative disorders [14,15]. With advances in gut–brain research investigations, probiotics that influence gut microbiome balance offer promising avenues for combating AD and dementia. Dairy products, including milk and milk-derived products, contain various nutrients such as proteins, calcium, magnesium, zinc, and vitamins, which are beneficial for physiological functions. Multiple investigations have indicated the beneficial effects against osteoporosis, depression, diabetes, and cardiovascular diseases [16,17,18]. Previous studies indicated that milk components, such as fats, proteins, casein, and whey protein, serve as substrates for beneficial gut bacteria, which produce bioactive peptides with diverse physiological effects [16,17]. However, the precise mechanism by which milk constituents positively affect gut health and cognitive function is unclear, prompting further investigation into the efficacy of milk-based therapies in improving cognitive function. Therefore, developing a probiotic formulation enriched with milk components is essential to harness their synergistic effects on gut health. This combination may enhance the gut–brain axis pathway, ultimately improving cognitive function. This randomized, double-blind, parallel-group clinical trial aimed to evaluate the effects of MH-Pro, a product prepared with a probiotic *Lacticaseibacillus rhamnosus* CBT LR5 and skim milk (non-fat dry milk), on cognitive function and physical changes in elderly individuals suspected of having MCI. The positive findings of this investigation contribute to the development of natural treatments with minimum or no side effects while significantly lowering treatment costs. 

## 2. Materials and Methods

### 2.1. Test Product

The MH-Pro test product was prepared with LR5, skim milk, maltose, dextrose, milk flavor, and starch. LR5 is a probiotic strain isolated from Korean human feces and classified as the KCTC 12202BP strain. Each packet of MH-Pro contains 5 × 10^9^ CFU of LR5 with 811 mg (low dose) or 2027.5 mg (high dose) of skim milk.

### 2.2. Study Design

**Figure 1:**
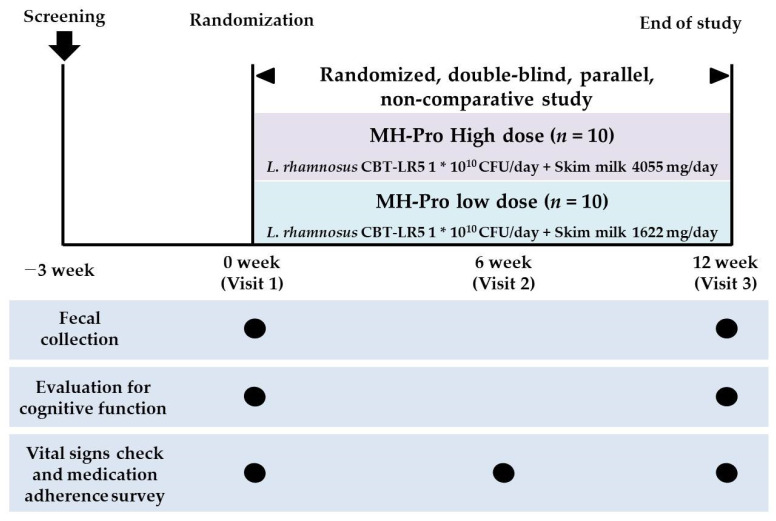
A schematic representation of the trial design.

This randomized, double-blind, parallel-group, non-comparative clinical trial was conducted from September 2023 to February 2024 at the Clinical Trial Center for Functional Foods (CTCF2). The clinical trial received IRB approval from Jeonbuk National University Hospital (approval No. CUH 2023-05-055) and is registered with the Clinical Research Information Service of the Republic of Korea (approval number: KCT0009966). The trial aimed to evaluate the efficacy and safety of MH-Pro at low (1 × 10^10^ CFU/day of LR5 and 1622 mg/day of skim milk) and high (1 × 10^10^ CFU/day of LR5 and 4055 mg/day of skim milk) doses in adults over 60 years with indications of MCI. In total, 20 subjects with low total scores on the AD assessment scale for the Korean version of the Consortium to Establish a Registry for Alzheimer’s Disease Assessment Packet (CERAD-K) and the Montreal Cognitive Assessment (MOCA-K), indicative of MCI, were included in the study. All the participants gave informed consent prior to the trial. Following consent, the subjects were screened and attended their first visit within three days of the screening visit to confirm eligibility based on the inclusion and exclusion criteria before being enrolled in the trial. All the participants were randomly assigned to one of two groups: the low-dose MH-Pro group or the high-dose MH-Pro group, with 10 participants in each group, at a 1:1 ratio. All participants received instructions to adhere to their regular lifestyle, including dietary practices and physical activity, and to abstain from consuming any additional functional foods, probiotics, or dietary supplements throughout the intervention. The 12-week intervention was designed on the basis of multiple clinical trials involving probiotics [19,20,21,22]. Furthermore, previous studies have confirmed the efficacy of LR5 probiotic supplementation for non-alcoholic fatty liver disease (NAFLD) through a 12-week clinical trial, supporting the intervention period [23,24]. A schematic representation of the study design is shown in Figure 1.

### 2.3. Inclusion and Exclusion Criteria

The inclusion criteria were: (1) men and women aged 60 or older who complained of memory decline during the screening test; (2) participants who met at least one of the following criteria, based on the three components of the CERAD-K [25] word list: a memory test score, a recall test score, or a recognition test score that fell 1 to 1.5 standard deviations (SD) below the normal range; (3) individuals with a total score of 22 or below on the MOCA-K; and (4) written informed consent obtained after a comprehensive explanation of the study and agreement to adhere to the study’s requirements.

Exclusion criteria were: (1) those undergoing treatment for major depressive disorder, having a history of treatment within the past 3 years, or a Beck Depression Inventory-II (BDI-II) score of 16 or higher; (2) individuals with conditions associated with cognitive impairment, such as dementia, Parkinson’s disease, or cerebral infarction; (3) individuals regularly consuming probiotics or fermented dairy products (e.g., yogurt, cheese) at least four times per week within 4 weeks prior to the screening test; (4) alcohol abuse or dependence within 3 months prior to the screening test; (5) individuals unable to read or understand the Korean language; (6) clinically significant acute or chronic diseases requiring treatment, including cardiovascular, cerebrovascular, endocrine, immune, respiratory, hepatobiliary, renal and urological, neuropsychiatric, musculoskeletal, inflammatory, hematologic/oncologic, or gastrointestinal disorders; (7) individuals on medications, health supplements to improve cognitive function or memory improvement, or prohibited concurrent drugs, within 1 month prior to the screening test; (8) those on psychotropic medications, including antipsychotics, antidepressants, mood stabilizers, hypnotics, or anxiolytics, within 1 month prior to the screening test; (9) those who had taken antibiotics within 4 weeks prior to the screening test; (10) those who had donated whole blood within 1 month or blood components within 2 weeks prior to the screening test; (11) those who had participated in other human clinical trials within 3 months prior to the screening test; (12) individuals with liver enzyme levels exceeding three times the upper limit of the reference range for AST or ALT, or a serum creatinine level > 2.0 mg/dL in diagnostic laboratory tests; (13) individuals deemed unsuitable for study participation by the principal investigator due to diagnostic laboratory test results or other reasons.

### 2.4. Randomization and Blinding

The volunteers selected for the trial were randomly assigned to the different experimental groups. Briefly, randomization was performed using a sequence generated in SAS^®^ (version 9.2, SAS Institute, Cary, NC, USA) and assigned sequentially to participants to ensure unbiased group allocation. Randomization codes were generated before the trial and provided to the clinical research institution. Later, the participants were randomly assigned in a 1:1 ratio to either the low-dose or high-dose MH-Pro group, with 10 participants in each. All participants completed the baseline evaluation at the first visit.

### 2.5. Assessment for Cognitive Function and Biochemical Parameters

The subjects underwent Alzheimer’s Disease Assessment Scale-Cognitive-13 (ADAS-Cog13) and MOCA-K assessments at baseline and at Week 12. Differences between the groups were tested using a non-parametric Wilcoxon signed-rank test with a significance level of less than 5%. The effects of the MH-Pro on cognitive functioning were evaluated by measuring changes in brain-derived neurotrophic factor (BDNF), amyloid-beta, acetylcholinesterase (AChE), prostaglandin E2 (PGE2), tau protein, and monocyte chemoattractant protein-1 (MCP-1) in the serum at before and after consumption. Differences between groups were tested using paired/independent *t*-tests with a significance level of less than 5%.

### 2.6. Fecal Sample Collection

Fecal samples were collected at baseline and after the intervention. To analyze the gut microbiota of the participants, the investigator provided fecal collection kits to the participants during their assessment visits at baseline (Visit 1) and the follow-up visit (Visit 3). Participants were instructed to collect approximately 3 g of feces in a collection container (1 container). Gut microbiota analysis was conducted using next-generation sequencing (NGS) and metagenomic analysis techniques to analyze the microbial community in the fecal samples comprehensively. All collected fecal samples were labeled with a unique code number generated using the SAS^®^ system to ensure proper identification and confidentiality. All the collected samples were stored at −80 °C until further processing.

### 2.7. Fecal DNA Extraction and Next-Generation Sequencing (NGS) Analysis

Microbial DNA was extracted using the SPINeasy DNA Kit for Feces/Soil (MP Biomedicals, Santa Ana, CA, USA) by following the manufacturer’s protocol. Briefly, the V4–V5 region of the bacterial 16S rRNA gene was amplified using specific primers: a forward primer (CCA GCM GCC GCG GTA ATW C) targeting the V4 region and a reverse primer (CC GTC AAT TYY) targeting the V5 region. The PCR products were purified with Agencourt^®^ AMPure XP beads (Beckman Coulter, Indianapolis, IN, USA) following the manufacturer’s guidelines. The isolated microbial DNA quality was evaluated using both the QuickDrop spectrophotometer (Molecular Devices, San Jose, CA, USA) and the Qubit™ dsDNA BR Assay Kit (Thermo Fisher Scientific, Waltham, MA, USA) to ensure quality and concentration for subsequent analyses. The quality of the sequencing libraries was confirmed using a 2100 Bioanalyzer (Agilent, Santa Clara, CA, USA), and sequencing was performed on the MiSeq platform (Illumina, San Diego, CA, USA) with the MiSeq Reagent Kit V2 (Illumina, San Diego, CA, USA). The sequencing data were processed into FASTQ files for downstream analysis. Preprocessing was performed using the Quantitative Insights into Microbial Ecology (QIIME 2, version 2023.5, http://qiime2.org, accessed on 21 August 2024) software suite. Noise reduction was performed with the DADA2 denoising method, and bacterial taxonomy was assigned using the Greengenes2 database. Most analytical visualizations were carried out with R (version 4.3.2). Spearman’s rank correlation was utilized for correlation analysis. The datasets were statistically compared using the Wilcoxon rank-sum test, Wilcoxon signed-rank test, and permutational multivariate analysis of variance (PERMANOVA).

### 2.8. Dietary Intake and Physical Activity Assessment

The dietary intake of the study participants was assessed using the dietary records method. Participants were provided with dietary record forms at the first visit (baseline; Week 0) and third visit (endpoint, Week 12). Participants were advised to document all foods consumed a day before the baseline (Week 0) and third visit (Week 12) as comprehensively as possible. The collected information on dietary intake was analyzed using the Can-Pro 4.0 software program (The Korean Nutrition Society, Seoul, Republic of Korea) to calculate the average daily intake. Furthermore, to evaluate changes in physical activity, the Global Physical Activity Questionnaire (GPAQ) was administered at the first visit (baseline; Week 0) and the third visit (Week 12) [26]. The metabolic equivalent of task (MET) value was calculated based on GPAQ data. Participants were instructed to maintain their daily lifestyle, diet, and physical activity throughout the study period.

### 2.9. Safety Outcome Measurements

The safety of study participants was evaluated before and after their participation in the clinical trial. Adverse events (AEs) and clinical conditions were monitored and documented in the case report forms. General parameters such as electrocardiograms (ECGs), blood pressure, pulse rate, white blood cells (WBCs), red blood cell (RBCs), hemoglobin, hematocrit, platelet count, neutrophils, lymphocytes, monocytes, eosinophils, basophils, and creatinine were analyzed as part of the basic safety assessment. Further, biochemical tests such as total bilirubin, total protein, alkaline phosphatase (ALP), gamma-glutamyl transferase (GGT), alanine transaminase (ALT), aspartate transaminase (AST), blood urea nitrogen (BUN), glucose, and creatinine were measured to assess the liver and kidney function. Additionally, total cholesterol, triglycerides, high-density lipoprotein cholesterol (HDL-C), low-density lipoprotein cholesterol (LDL-C), and non-HDL cholesterol were analyzed to determine the status of lipid metabolism. These comprehensive evaluations ensured the health and safety of participants throughout the study.

### 2.10. Statistical Analysis

All statistical analyses were conducted using SAS^®^ version 9.4 (SAS Institute, Cary, NC, USA). Continuous variables are presented as the mean ± standard deviation (SD), while categorical variables are expressed as n (%). The final results were evaluated using the full analysis set (FAS). Baseline characteristics of the MH-Pro high-dose group and the MH-Pro low-dose group were compared using independent *t*-tests for continuous variables and Chi-square tests (or Fisher’s exact tests) for categorical variables. Comparisons within groups (before and after the 12-week intervention) were analyzed using paired *t*-tests or Wilcoxon signed-rank tests, depending on the data distribution. Independent *t*-tests were performed to test for homogeneity of baseline characteristics between the two groups for efficacy endpoints. If the baseline results differed significantly between groups, analysis of covariance (ANCOVA) was used, with the baseline values included as covariates, to compare post-intervention outcomes between groups. For efficacy endpoints, normality tests were performed. If normality was severely violated, non-parametric tests such as the Wilcoxon rank-sum test or the Wilcoxon signed-rank test were applied. Statistical significance was determined at the *p* < 0.05 level.

## 3. Results

### 3.1. Participant Recruitment and General Demographic Characteristics

**Figure 2:**
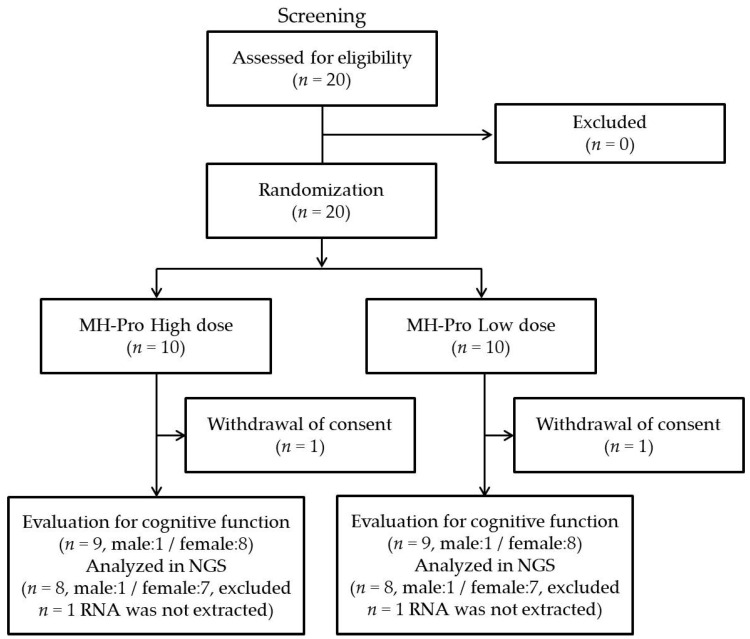
Schematic representation of the selection and distribution of participants in the trial.

Participants were recruited from individuals visiting the CTCF2 at Jeonbuk National University Hospital, Jeonju, Republic of Korea. All participants provided informed consent, which included signatures from either themselves or their family members, before participating in the study. In total, 20 subjects over 60 years were recruited for screening, and 20 were selected on the basis of the set inclusion and exclusion criteria. All participants underwent assessments related to AEs and compliance during the second and third visits. All participants exhibited a high compliance rate of over 97%, and no AEs were reported during the intervention. The participants’ mean age was 68.95 ± 3.05, and all were experiencing MCI as per the criteria established by CERAD-K and MOCA-K (Figure 2). All the demographic information such as sex, age, education, height, weight, BMI, systolic/diastolic blood pressure, pulse rate, alcoholic status, smoking status, CERAD-K score, MOCA-K score, and BDI score of participants is shown in Table 1. The MH-Pro high and MH-Pro low dose groups showed no significant differences among the measured demographic characters.

### 3.2. Primary/Secondary Outcomes for Cognitive Function

This investigation met the primary and secondary endpoints after 12 weeks of MH-Pro consumption. After 12 weeks, a significant difference was observed in the ADAS-Cog13 concentration test in the MH-Pro high-dose group (*p* = 0.04). Collectively, no statistical differences were observed between MH-Pro high- and low-dose participants (Table 2). In addition, for the MOCA-K, the naming and delayed recall domains showed a significant improvement in the MH-Pro low-dose group (*p* = 0.01 and *p* = 0.0039, respectively). Specifically, the MH-Pro low-dose group performed significantly better on the delayed recall test. Moreover, orientation was significantly improved in the MH-Pro high-dose group (*p* = 0.05). Together, the low-dose group demonstrated a significant cognitive improvement in total (*p* = 0.004), while the MH-Pro low-dose group participants showed a trend towards improvement (*p* = 0.06) (Table 3). Additionally, a significant reduction in levels of amyloid*β*-1 40/42 (*p* = 0.03) was observed in the MH-Pro low-dose group, indicating meaningful changes in cognitive-related blood markers (Table 4).

### 3.3. Gut Microbiota Analysis

**Figure 3:**
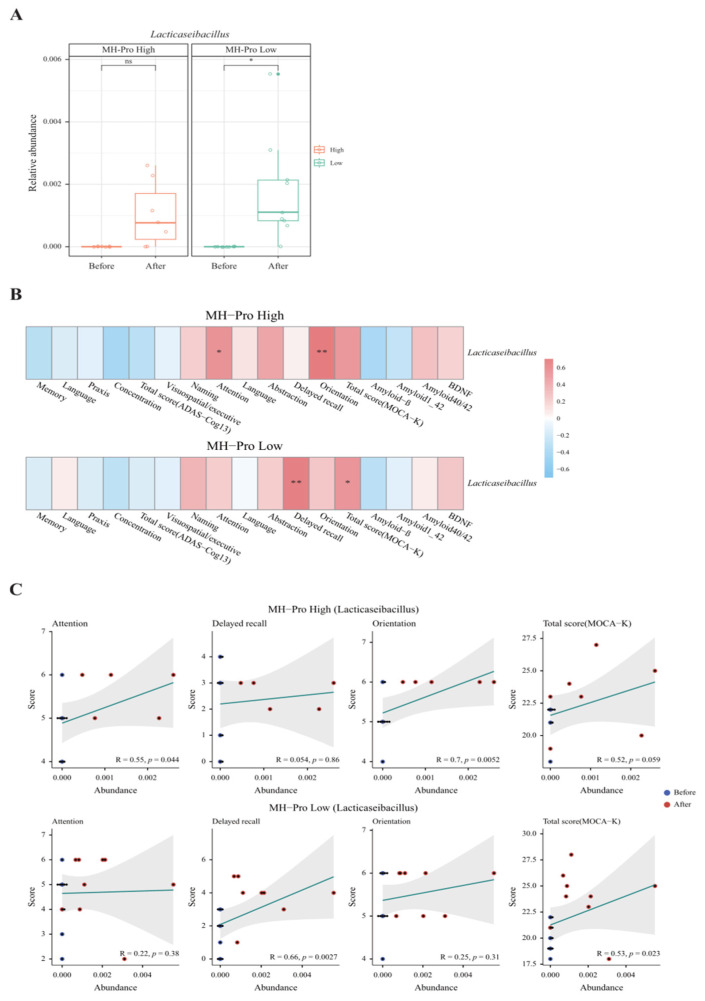
Impact of MH-Pro supplementation on *Lacticaseibacillus* abundance and cognitive function. (**A**) Relative abundance of *Lacticaseibacillus* before and after supplementation with MH-Pro. (**B**) Correlation heatmap showing the association between the relative abundance of *Lacticaseibacillus* and various cognitive function parameters in the MH-Pro high-dose and MH-Pro low-dose groups. (**C**) Scatter plots showing the correlation between the relative abundance of *Lacticaseibacillus* and specific cognitive function scores (attention, delayed recall, orientation, and total MoCA-K score) in the MH-Pro high-dose and MH-Pro low-dose groups. “ns” indicates non-significance, while * *p* < 0.05 and ** *p* < 0.01 represent statistically significant differences at 5 and 1%, respectively. The strength and direction of correlations are indicated by the corresponding R-values, with statistical significance denoted by the accompanying *p*-values.

Fecal samples were assessed for relative composition at the phylum and genus levels to observe overall changes in taxa composition. Assessment of fecal samples collected at baseline and post-intervention revealed significant changes in bacterial composition (Figure S1). Specifically, *Lacticaseibacillus* was significantly increased in the MH-Pro low-dose group (*p* = 0.01) (Figure 3A). Interestingly, a correlation analysis between *Lacticaseibacillus* abundance and MOCA-K scores revealed positive and statistically significant associations. Further, the MH-Pro high-dose group demonstrated positive and significant correlations with attention (*p* = 0.04) and orientation (*p* = 0.005), whereas the MH-Pro low-dose group showed significant associations with delayed recall (*p* = 0.003) and total score (*p* = 0.02) (Figure 3B). Further detailed analysis of these four metadata categories demonstrated statistical significance (Figure 3C). Alpha and beta diversity analyses were performed for each group at baseline and post-intervention. However, alpha diversity analyses revealed that supplementation of MH-Pro at high or low doses did not significantly alter the number of taxa or the relative abundance of those taxa. Similarly, beta diversity showed no significant difference in microbial composition between the groups (Figure S2).

### 3.4. Dietary Intake and Physical Activity

Diet significantly influences cognitive abilities, and adding skim milk with probiotics enhances cognitive performance. Thus, the diet of participants during the intervention and their adherence to the supplementation schedule was critical to the investigation. In the MH-Pro high-dose group, daily calorie, carbohydrate, and lipid consumption significantly increased after 12 weeks (*p* < 0.05), although no significant changes were observed between the MH-Pro high- and low-dose groups (*p* > 0.05). Additionally, the intake of protein and dietary fiber showed no statistically significant differences between the two groups (*p* > 0.05). Also, the metabolic equivalent of task (MET) values showed no significant differences within or between the two groups (*p* > 0.05), indicating consistent levels of physical activity among the trial participants. The dietary intake of the subjects in the MH-Pro high- and low-dose groups during the 12-week intervention period is shown in Table S1.

### 3.5. Safety and Adverse Events

No AEs were observed during the intervention period, and no significant changes or differences were observed in the safety parameters, including laboratory tests, ECG, and vital signs (BP and pulse) (*p* > 0.05). In the laboratory tests, creatine kinase (CK) levels increased in the MH-Pro high-dose group, while it decreased in the MH-Pro low-dose group, indicating a significant difference between the two groups. However, CK levels in both groups remained within the normal range, confirming their safety. Other laboratory test parameters were within standard normal ranges (Table S2). Collectively, the intervention displayed a positive safety profile, with no AEs and all safety parameters, including CK levels, being consistently within normal ranges throughout the intervention.

## 4. Discussion

The study presents a novel approach and is the first to demonstrate cognitive improvement with supplementation of MH-Pro in elderly individuals suspected of having MCI through a randomized, double-blind, parallel-design, non-comparative trial. The participants’ mean age in the investigations was 68.95 ± 3.05, with mild cognitive impairment. This age group is believed to be ideal for enhancing memory before significant frailty or dementia develops. In this investigation, all participants showed a compliance rate of over 97%, with no AEs related to the intervention, strengthening the trial’s validity, and strongly supporting the safety of MH-Pro. Interestingly, lack of side effects appeared to be consistent with the safety profile of most probiotics except in critically ill patients in intensive care units, critically sick infants, and postoperative patients [27]. Probiotic strains like *L. rhamnosus* are generally recognized as safe [28,29,30], and the applied doses were based on a previous investigation which confirmed its efficacy and potential safety characteristics. This study’s observations were in alignment with the existing literature on probiotics, reinforcing their safety in cognitive function interventions.

The ADAS-Cog13 and MoCA-K tests are essential for evaluating cognitive function, since they offer superior sensitivity, specificity, and diagnostic accuracy for identifying cognitive impairments. Specifically, ADAS-Cog13 is highly recommended for its efficacy [31] and is used in over 40 countries for MCI diagnosis. Moreover, the MOCA-K, adapted for the Korean population to address linguistic differences, is highly appropriate for the current investigation to identify MCI.

In this trial, the greater efficacy observed in the MH-Pro low-dose group compared with the high-dose group suggests that the beneficial effects of skim milk are optimized at lower doses (1622 mg/day) rather than higher doses (4055 mg/day). This specific observation has been described earlier, which reported that multiple clinical trials suggest that moderate milk consumption (e.g., 1–2 servings per day) has a beneficial effect on cognitive function, while excessive milk consumption shows no on brain function [32]. It is well known that proline and *β*-lactoglobulin are the prominent proteins in skim milk. Also, skim milk supports probiotic survival and the growth of starter microorganisms [18,33,34,35,36]. Previous trials demonstrated that milk components, like fat, protein, casein, and whey protein, can promote the growth of beneficial gut bacteria and produce bioactive peptides that may influence cognitive function [16,17,18,37].

A clinical study by Bilikiewicz and Gaus, which involved patients over 50 years old suffering from probable AD, indicated that the intake of proline-rich colostrum resulted in significant improvements in cognitive functions (memory, language, reasoning) [38]. In another clinical study by Masahiro Kita et al., which examined the cognitive function improvement effects of whey protein rich in *β*-lactolin, it was found that the *β*-lactotripeptide derived from *β*-lactoglobulin positively impacted the dorsolateral prefrontal cortex (DLPFC) of the brain, which is related to language fluency functions [39]. Moreover, the meta-analysis of milk consumption by Wu et al. showed that milk intake can decrease the risk of cognitive disorders [40]. Another investigation of full-fat milk intake indicated that the consumption of full-fat milk is associated with reduced cognitive function and an elevated risk of MCD [41]. These observations strongly support the study findings on using skim milk at lower doses.

Moreover, previous clinical research linked to memory and language is thought to be closely related to the efficacy in naming and delayed recall of the MOCA-K test conducted in this clinical study. These investigations also strongly support the observations of this study. Further, recent studies on the gut–brain axis suggest that various metabolic products of the gut microbiota, including short-chain fatty acids (SCFAs) [42,43,44], G protein-coupled receptors [45], neurotransmitters [46], and neurotrophic factors [47], indicated their crucial role in regulating cognitive function. For instance, SCFAs are known to maintain the integrity of the blood–brain barrier and may affect cognitive processes [48]. Also, SCFAs can interfere with amyloid-beta plaque formation by disrupting protein–protein interactions [49], which explains the observed reduction in amyloid-β1 40/42 levels observed in this study. The interaction between the gastrointestinal system and the brain has been recognized for a while, including direct neurological impulses as well as indirect hormonal and enzymatic linkages [50,51,52,53]. These developments paved the way for using probiotics as an innovative, natural treatment against illness associated with the brain. Also, the use of probiotics offers minimal side effects. Therefore, regulating the gut–brain axis potentially contributes to improvements in cognitive and brain functions. In this investigation, MH-Pro, containing LR5 strain supplementation over 12 weeks, significantly improved the *Lacticaseibacillus* sp. in the gut of the participants. Specifically, the LR5 strain, isolated and patented by Cell Biotech Co., Gimpo-si, Republic of Korea, has been reported to enhance gut function, combat obesity, and improve immune responses [23].

In particular, in a previous in vivo study, oral supplementation of MH-Pro on scopolamine-induced cognitively impaired mice demonstrated enhanced cognitive function. The passive avoidance test (PAT) results showed that latency time was significantly increased. Moreover, in the novel object recognition test (NORT), the time spent exploring the novel object was significantly increased. Additionally, we observed a significant reduction in the levels of the inflammation-related markers PGE2 and TNF-*α* in the serum. These findings were reflected in this clinical trial. Interestingly, ingestion of *Lacticaseibacillus rhamnosus* Fmb14 is known to prevent depression-like behavior and brain neural activity by regulating the gut–brain axis in colitis mice [54]. These observations are associated with the observations with attention and delayed recall parameters of the MoCA-K, as well as the language and concentration parameters of the ADAS-Cog13 assessment of the study. Moreover, correlation analyses related to cognition showed significant associations with the MOCA-K total score, attention, orientation, and delayed recall.

Furthermore, NGS, a high-throughput method for analyzing the gut microbiome, revealed that MH-Pro supplementation positively influenced the gut microbiome environment and may have regulated the gut–brain axis, potentially enhancing brain function. Collectively, this study’s observations and previous reports suggest the involvement of the gut–brain axis in improving cognitive function. However, the study lacks a comprehensive understanding of the mechanism of action of MH-Pro. Previous reports demonstrated that *Lacticaseibacillus rhamnosus* can alter the gut microbiome’s composition by enhancing the production of bioactive compounds such as short-chain fatty acids (SCFAs) [10] and neurotransmitters [55].

On the other hand, skim milk is rich in bioactive peptides such as casein-derived peptides, which can interact with gut receptors and influence gut permeability and immune responses [56,57]. Also, milk peptides directly affect brain signaling pathways by influencing the synthesis of neurotransmitters. These alterations in gut barrier function, the synthesis of neurotransmitters, and systemic inflammation modulate the gut–brain axis, positively affecting neurocognitive processes. Thus, the synergistic effects of probiotics and milk components potentially improve cognitive function by optimizing gut health and reducing neuroinflammation through the gut–brain axis.

This trial has few limitations that may impact its overall applicability. However, this study serves as a crucial step in the development of natural treatments against cognitive function with minimal or no side effects. This study was developed and designed as a pilot investigation, considering the challenges associated with recruiting individuals over 60 with MCI. Thus, the number of participants in the study is relatively small. Moreover, due to feasibility considerations, the recruitment process did not include a comprehensive neuropsychological assessment. However, this approach aligns with previous studies of similar scope and ensures valid and reliable subject classification. Additionally, there was a gender imbalance, with both groups predominantly consisting of females. However, previous studies indicate that cognitive impairment is more prevalent in the elderly population, particularly among females [58]. This higher occurrence can be attributed to various factors, including hormonal influences and lifestyle differences [32,59,60].

The gender imbalance, though it may seem like a limitation, is outweighed by the increased risk in women, making their greater representation advantageous for the study’s relevance and findings. Moreover, high education potentially reduces the risk of MCI and AD [61]. In this study, the mean education level of participants was 12 years which may be considered relatively high education. However, considering the standard deviation, the study participants are from a broad educational range, from middle school to university-level education. This distribution ensures that the study is not limited to highly educated individuals but rather represents a diverse cognitive profile. Also, not all studies link lower education to a greater risk of cognitive impairments like dementia [62]. Further, recent studies suggested that the analysis of p-tau and APOE genotypes in human plasma could serve as reliable biomarkers for cognitive assessments and the predictive diagnosis of AD [63,64,65,66]. Therefore, building on this pilot investigation, further studies will be conducted on a larger scale and for an extended period, with the inclusion of biomarker evaluation such as p-tau217 and APOE4. Additionally, this study did not include individuals below 60 years, an age group that may represent the preclinical stage for preventive interventions. However, by focusing on individuals aged 60 and above with MCI, who are already experiencing early-stage cognitive decline, this study aligns more closely with early intervention rather than primary prevention. This approach provides valuable insights into potential therapeutic effects at a stage where intervention may still influence disease progression. Interestingly, the trial offers several advantages compared with some similar previous clinical studies. First, unlike the large-scale Finnish Geriatric Intervention Study to Prevent Cognitive Impairment and Disability (FINGER), which was conducted across multiple countries, this study is smaller in scale but focuses on a single population in Asia. This helps better generalize the findings to the specific population or ethnicity [67,68]. Additionally, the study includes only dietary modifications, which are relatively more straightforward to adopt than other investigations requiring multiple lifestyle changes. This method is particularly advantageous for the elderly, who may find it challenging to adhere to extensive lifestyle modifications. This investigation’s compliance rate was over 97%, with no AEs related to the intervention, supporting its straightforward approach.

## 5. Conclusions

The trial’s findings revealed the definitive effect of MH-Pro in high and low doses. The low-dose group showed comprehensive cognitive enhancement, whereas the high-dose group showed a positive tendency toward improvement. Specifically, naming and delayed recall in the MH-Pro low-dose group significantly improved, while orientation was improved in the MH-Pro high-dose group. Also, a significant decrease in amyloid-β1-40/42 levels with MH-Pro supplementation indicated cognitive improvement. Collectively, this study provides foundational evidence to recognize the use of LR5 and skim milk to prepare a probiotic supplement that enhances cognitive function in the aging population.

## Figures and Tables

**Table 1:** General demographic characteristics of the study subjects.

		MH-Pro High Dose (*n* = 10)	MH-Pro Low Dose (*n* = 10)	Total (*n* = 20)	*p*-Value ^(1)^
Sex	Male	1 (10.0)	1 (10.0)	2 (10.0)	>0.99 ^(3)^
	Female	9 (90.0)	9 (90.0)	18 (90.0)	
Age (years)		68.50 ± 2.59	69.40 ± 3.53	68.95 ± 3.05	0.52
Education (years)		12.30 ± 3.33	11.70 ± 2.98	12.0 ± 3.09	0.68
Height (cm)		156.20 ± 4.94	153.80 ± 5.69	155.0 ± 5.33	0.33
Weight (kg)		58.25 ± 5.11	58.42 ± 7.22	58.34 ± 6.09	0.95
BMI (kg/m^2^)		23.86 ± 1.45	24.66 ± 2.45	24.26 ± 2.0	0.39
Systolic blood pressure (mmHg)		125.20 ± 15.75	129.60 ± 15.39	127.40 ± 15.32	0.54
Diastolic blood pressure (mmHg)		74.0 ± 9.30	75.10 ± 9.52	74.55 ± 9.17	0.80
Pulse rate (beats/min)		72.20 ± 10.02	72.20 ± 8.97	72.20 ± 9.25	>0.99
Alcoholic drinking status, n (%)		2 (20.0)	2 (20.0)	4 (20.0)	>0.99 ^(2)^
Amount of alcohol status (g/week)		8.0 ± 8.49	7.20 ± 5.09	7.60 ± 5.73	0.92
Smoking status, n (%)		0 (0.0)	0 (0.0)	0 (0.0)	-
Comorbidities, n (%)	Hypertension	5 (35.7)	2 (14.3)	7 (50.0)	0.50 ^(3)^
	Dyslipidemia	4 (28.6)	3 (21.4)	7 (50.0)	
Medications, n (%)	Hypertension	7 (41.2)	3 (21.4)	10 (32.3)	0.30 ^(3)^
	Dyslipidemia	4 (23.5)	4 (28.6)	8 (25.8)	
	Vaccination (COVID-19, influenza)	2 (11.8)	0 (0.0)	2 (6.4)	
	None	4 (23.5)	7 (50.0)	11 (35.5)	
CERAD-K (score)	Word list memory	19.60 ± 2.41	19.40 ± 2.88	19.50 ± 2.59	0.87
	Word list recall	5.70 ± 1.34	5.90 ± 0.88	5.80 ± 1.11	0.70
	Word list recognition	8.20 ± 1.03	7.90 ± 0.99	8.05 ± 1.0	0.52
MOCA-K	Total score (score)	20.30 ± 1.89	19.70 ± 1.64	20.0 ± 1.75	0.46
BDI (score)		9.20 ± 3.79	8.20 ± 4.42	8.70 ± 4.04	0.59

Values are presented as the mean ± SD or n (%). ^(1)^ Analyzed by the independent *t*-test; ^(2)^ analyzed by chi-square test; ^(3)^ analyzed by Fisher’s exact test. Abbreviations: BMI, body mass index; CERAD-K, A Korean version of the Consortium to Establish a Registry for Alzheimer’s Disease Assessment Packet; MOCA-K, Korean Montreal Cognitive Assessment; BDI, Beck Depression Inventory.

**Table 2:** Assessment of cognitive changes via the ADAS-Cog13 test.

		MH-Pro High Dose (*n* = 9)	MH-Pro Low Dose (*n* = 9)	*p*-Value ^(2)^
Memory (score)	Baseline	11.44 ± 4.72	13.44 ± 3.43	0.32
	Visit 3	10.78 ± 2.59	11.44 ± 3.84	0.39
	Change from baseline	−0.67 ± 3.43	−2.0 ± 3.0	
	*p*-value ^(1)^	0.58	0.08	
Language (score)	Baseline	1.89 ± 1.27	1.67 ± 1.50	0.74
	Visit 3	0.89 ± 0.93	1.33 ± 1.0	0.42
	Change from baseline	−1.0 ± 1.41	−0.33 ± 1.94	
	*p*-value ^(1)^	0.067	0.62	
Praxis (score)	Baseline	1.33 ± 0.71	1.89 ± 1.36	0.29
	Visit 3	1.33 ± 0.71	1.67 ± 0.87	0.72
	Change from baseline	0.0 ± 0.71	−0.22 ± 1.64	
	*p*-value ^(1)^	>0.99	0.70	
Concentration (score)	Baseline	2.11 ± 1.05	1.67 ± 0.71	0.31
	Visit 3	1.33 ± 0.87	1.33 ± 0.87	0.32
	Change from baseline	−0.78 ± 0.97	−0.33 ± 0.87	
	*p*-value ^(1)^	0.04	0.28	
Total score (score)	Baseline	16.78 ± 6.46	18.67 ± 3.87	0.46
	Visit 3	14.33 ± 3.32	15.78 ± 4.68	0.83
	Change from baseline	−2.44 ± 4.13	−2.89 ± 4.37	
	*p*-value ^(1)^	0.11	0.08	

Values are presented as the mean ± SD. ^(1)^ Analyzed by the paired *t*-test; ^(2)^ analyzed by the independent *t*-test.

**Table 3:** Assessment of cognitive changes using the MOCA-K screening test.

Variables (Score)		MH-Pro High Dose (*n* = 9)	MH-Pro Low Dose (*n* = 9)	*p*-Value ^(2)^
Visuospatial/ executive	Baseline	3.22 ± 1.09	3.56 ± 0.73	0.46
	Visit 3	3.56 ± 1.01	3.67 ±0.87	0.63
	Change from baseline	0.33 ± 1.0	0.11 ± 0.93	
	*p*-value ^(1)^	0.35	0.73	
Naming	Baseline	2.89 ± 0.33	2.33 ± 0.71	0.06
	Visit 3	3.0 ± 0.0	2.89 ± 0.33	0.05
	Change from baseline	0.11 ± 0.33	0.56 ±0.53	
	*p*-value ^(1)^	0.35	0.01	
Attention	Baseline	4.44 ± 1.01	4.44 ± 1.24	>0.99
	Visit 3	5.44 ± 0.73	4.89 ± 1.36	0.43
	Change from baseline	1.0 ± 1.41	0.44 ± 1.51	
	*p*-value ^(1)^	0.07	0.40	
Language	Baseline	1.78 ± 0.67	2.0 ± 0.71	0.50
	Visit 3	1.89 ± 0.93	2.0 ± 0.71	0.81
	Change from baseline	0.11 ± 0.78	0.0 ± 1.12	
	*p*-value ^(1)^	0.68	>0.99	
Abstraction	Baseline	1.0 ± 0.50	0.78 ± 0.44	0.33
	Visit 3	1.0 ± 0.71	1.22 ± 0.67	0.15
	Change from baseline	0.0 ±0.50	0.44 ± 0.73	
	*p*-value ^(1)^	>0.99	0.10	
Delayed recall	Baseline	2.22 ±1.30	1.56 ± 1.33	0.30
	Visit 3	2.56 ±1.24	3.56 ±1.33	0.03
	Change from baseline	0.33 ± 1.41	2.0 ± 1.50	
	*p*-value ^(1)^	0.50	0.009	
Orientation	Baseline	5.11 ± 0.60	5.33 ± 0.71	0.48
	Visit 3	5.78 ± 0.44	5.56 ± 0.53	0.24
	Change from baseline	0.67 ± 0.87	0.22 ± 0.67	
	*p*-value ^(1)^	0.05	0.35	
Total score	Baseline	20.67 ± 1.58	20.0 ± 1.41	0.36
	Visit 3	23.22 ± 2.49	23.78 ± 2.91	0.42
	Change from baseline	2.56 ± 3.43	3.78 ± 2.77	
	*p*-value ^(1)^	0.06	0.004	

Values are presented as the mean ± SD. ^(1)^ Analyzed by the paired *t*-test; ^(2)^ analyzed by the independent *t*-test.

**Table 4:** The influence of MH-Pro on amyloid-*β* and BDNF in older adults suspected of MCI.

		MH-Pro High Dose (*n* = 9)	MH-Pro Low Dose (*n* = 9)	*p*-Value ^(2)^
Amyloid-*β*1–40 (pg/mL)	Baseline	205.94 ± 22.26	202.83 ± 39.67	0.84
	Visit 3	193.28 ± 15.41	196.04 ± 28.40	0.73
	Change from baseline	−12.66 ± 25.73	−6.79 ± 44.09	
	*p*-value ^(1)^	0.18	0.66	
Amyloid-*β* 1–42 (pg/mL)	Baseline	12.66 ± 6.98	9.28 ± 8.72	0.38
	Visit 3	12.33 ± 7.29	9.26 ± 5.14	0.88
	Change from baseline	−0.33 ± 3.83	−0.02 ± 4.47	
	*p*-value ^(1)^	0.80	0.99	
Amyloid-*β*1 40/42	Baseline	20.47 ± 10.0	39.16 ± 28.12	0.09
	Visit 3	20.30 ± 9.85	29.29 ± 18.70	0.04
	Change from baseline	−0.17 ± 6.88	−9.87 ± 10.98	
	*p*-value ^(1)^	0.94	0.02	
BDNF (pg/mL)	Baseline	30,055.56 ± 5669.46	27,200.0 ± 11,995.42	0.53
	Visit 3	30,800.0 ± 6957.73	32,255.56 ± 6427.89	0.31
	Change from baseline	744.44 ± 3354.52	5055.56 ± 11,698.94	
	*p*-value ^(1)^	0.52	0.23	

Values are presented as the mean ± SD. ^(1)^ Analyzed by the paired *t*-test; ^(2)^ analyzed by the independent *t*-test.

## Data Availability

The datasets generated during and/or analyzed during the current study are available from the corresponding author on reasonable request due to privacy.

## Supplementary Materials

The following supporting information can be downloaded at: https://www.mdpi.com/article/10.3390/nu17040691/s1, Figure S1. Relative abundance of gut microbiota at phylum and genus levels in MH-Pro high and low groups before and after supplementation. Figure S2. Alpha and beta diversity analyses of gut microbiota in MH-Pro high and low groups before and after supplementation. Table S1. Major nutrient intakes and metabolic equivalent values of the intervention groups measured at baseline and 12 weeks. Table S2. Laboratory profiles of the subjects in this study.

## References

[1] WHO (2024). Ageing and Health; Fact Sheets. https://www.who.int/news-room/fact-sheets/detail/ageing-and-health.

[2] Central Dementia Center (2024). Korean Dementia Observatory 2023.

[3] (2024). Alzheimer’s Association2024 Alzheimer’s disease facts and figures. Alzheimer’s Dement..

[4] Atri A. (2019). The Alzheimer’s disease clinical spectrum: Diagnosis and management. Med. Clin..

[5] Nanjiba R. (2024). An Updated Review on the Approved and Potential Drugs for the Treatment of Alzheimer’s Disease. Bachelor’s Thesis.

[6] Ameen T.B., Kashif S.N., Abbas S.M.I., Babar K., Ali S.M.S., Raheem A. (2024). Unraveling Alzheimer’s: The promise of aducanumab, lecanemab, and donanemab. Egypt. J. Neurol. Psychiatry Neurosurg..

[7] Lazarević-Pašti T. (2023). Side effects of Alzheimer’s disease treatment. Curr. Med. Chem..

[8] Martinez-Lopez S., Tabone M., Clemente-Velasco S., Gonzalez-Soltero M.D.R., Bailen M., de Lucas B., Bressa C., Dominguez-Balmaseda D., Marin-Munoz J., Antunez C. (2024). A systematic review of lifestyle-based interventions for managing Alzheimer’s disease: Insights from randomized controlled trials. J. Alzheimer’s Dis..

[9] Wu X., Zhang T., Tu Y., Deng X., Sigen A., Li Y., Jing X., Wei L., Huang N., Cheng Y. (2023). Multidomain interventions for non-pharmacological enhancement (MINE) program in Chinese older adults with mild cognitive impairment: A multicenter randomized controlled trial protocol. BMC Neurol..

[10] Markowiak-Kopeć P., Śliżewska K. (2020). The effect of probiotics on the production of short-chain fatty acids by human intestinal microbiome. Nutrients.

[11] Bock P.M., Telo G.H., Ramalho R., Sbaraini M., Leivas G., Martins A.F., Schaan B.D. (2021). The effect of probiotics, prebiotics or synbiotics on metabolic outcomes in individuals with diabetes: A systematic review and meta-analysis. Diabetologia.

[12] Qi D., Nie X.-L., Zhang J.-J. (2020). The effect of probiotics supplementation on blood pressure: A systemic review and meta-analysis. Lipids Health Dis..

[13] Lopez-Santamarina A., Gonzalez E.G., Lamas A., Mondragon A.d.C., Regal P., Miranda J.M. (2021). Probiotics as a possible strategy for the prevention and treatment of allergies. A narrative review. Foods.

[14] Chen X., D’Souza R., Hong S.-T. (2013). The role of gut microbiota in the gut-brain axis: Current challenges and perspectives. Protein Cell.

[15] Torres-Fuentes C., Schellekens H., Dinan T.G., Cryan J.F. (2017). The microbiota–gut–brain axis in obesity. Lancet Gastroenterol. Hepatol..

[16] Park Y.W., Nam M.S. (2015). Bioactive Peptides in Milk and Dairy Products: A Review. Korean J. Food Sci. Anim. Resour..

[17] Korhonen H. (2009). Milk-derived bioactive peptides: From science to applications. J. Funct. Foods.

[18] Aslam H., Marx W., Rocks T., Loughman A., Chandrasekaran V., Ruusunen A., Dawson S.L., West M., Mullarkey E., Pasco J.A. (2020). The effects of dairy and dairy derivatives on the gut microbiota: A systematic literature review. Gut Microbes.

[19] Chong H.X., Yusoff N.A.A., Hor Y.-Y., Lew L.-C., Jaafar M.H., Choi S.-B., Yusoff M.S.B., Wahid N., Abdullah M.F.I.L., Zakaria N. (2019). Lactobacillus plantarum DR7 alleviates stress and anxiety in adults: A randomised, double-blind, placebo-controlled study. Benef. Microbes.

[20] Liu Y., Yu X., Yu L., Tian F., Zhao J., Zhang H., Qian L., Wang Q., Xue Z., Zhai Q. (2021). Lactobacillus plantarum CCFM8610 alleviates irritable bowel syndrome and prevents gut microbiota dysbiosis: A randomized, double-blind, placebo-controlled, pilot clinical trial. Engineering.

[21] Sanborn V., Azcarate-Peril M.A., Updegraff J., Manderino L., Gunstad J. (2020). Randomized clinical trial examining the impact of *Lactobacillus rhamnosus* GG probiotic supplementation on cognitive functioning in middle-aged and older adults. Neuropsychiatr. Dis. Treat..

[22] Yang X., He X., Xu S., Zhang Y., Mo C., Lai Y., Song Y., Yan Z., Ai P., Qian Y. (2023). Effect of Lacticaseibacillus paracasei strain Shirota supplementation on clinical responses and gut microbiome in Parkinson’s disease. Food Funct..

[23] Ahn S.B., Jun D.W., Kang B.-K., Lim J.H., Lim S., Chung M.-J. (2019). Randomized, double-blind, placebo-controlled study of a multispecies probiotic mixture in nonalcoholic fatty liver disease. Sci. Rep..

[24] Choi J., Son D., An S., Cho E., Lim S., Lee H.-J. (2024). Effects of *Lactiplantibacillus plantarum* CBT LP3 and *Bifidobacterium breve* CBT BR3 supplementation on weight loss and gut microbiota of overweight dogs. Sci. Rep..

[25] Hwang Y.H., Park S., Paik J.W., Chae S.W., Kim D.H., Jeong D.G., Ha E., Kim M., Hong G., Park S.H. (2019). Efficacy and Safety of *Lactobacillus Plantarum* C29-Fermented Soybean (DW2009) in Individuals with Mild Cognitive Impairment: A 12-Week, Multi-Center, Randomized, Double-Blind, Placebo-Controlled Clinical Trial. Nutrients.

[26] Armstrong T., Bull F. (2006). Development of the World Health Organization Global Physical Activity Questionnaire (GPAQ). J. Public Health.

[27] Didari T., Solki S., Mozaffari S., Nikfar S., Abdollahi M. (2014). A systematic review of the safety of probiotics. Expert Opin. Drug Saf..

[28] Chen T., Shao Y., Zhang Y., Zhao Y., Han M., Gai Z. (2024). In vitro and in vivo genome-based safety evaluation of *Lacticaseibacillus rhamnosus* LRa05. Food Chem. Toxicol..

[29] Mathipa-Mdakane M.G., Thantsha M.S. (2022). *Lacticaseibacillus rhamnosus*: A Suitable Candidate for the Construction of Novel Bioengineered Probiotic Strains for Targeted Pathogen Control. Foods.

[30] Zhao L., Zhang Y., Liu Y., Zhong J., Zhang D. (2023). Assessing the Safety and Probiotic Characteristics of *Lacticaseibacillus rhamnosus* X253 via Complete Genome and Phenotype Analysis. Microorganisms.

[31] Skinner J., Carvalho J.O., Potter G.G., Thames A., Zelinski E., Crane P.K., Gibbons L.E., the Alzheimer’s Disease Neuroimaging Initiative (2012). The Alzheimer’s Disease Assessment Scale-Cognitive-Plus (ADAS-Cog-Plus): An expansion of the ADAS-Cog to improve responsiveness in MCI. Brain Imaging Behav..

[32] Janicki S.C., Schupf N. (2010). Hormonal influences on cognition and risk for Alzheimer’s disease. Curr. Neurol. Neurosci. Rep..

[33] Calvo M.V., Kohen V.L., Díaz-Mardomingo C., García-Herranz S., Baliyan S., Tomé-Carneiro J., Colmenarejo G., Visioli F., Venero C., Fontecha J. (2023). Milk fat globule membrane-enriched milk improves episodic memory: A randomized, parallel, double-blind, placebo-controlled trial in older adults. J. Funct. Foods.

[34] Ohsawa K., Uchida N., Ohki K., Nakamura Y., Yokogoshi H. (2015). *Lactobacillus helveticus*–fermented milk improves learning and memory in mice. Nutr. Neurosci..

[35] Ohsawa K., Uchida N., Ohki K., Yokogoshi H. (2018). Identification of peptides present in sour milk whey that ameliorate scopolamine-induced memory impairment in mice. Int. J. Food Sci. Nutr..

[36] Yeon S.-W., You Y.S., Kwon H.-S., Yang E.H., Ryu J.-S., Kang B.H., Kang J.-H. (2010). Fermented milk of *Lactobacillus helveticus* IDCC3801 reduces beta-amyloid and attenuates memory deficit. J. Funct. Foods.

[37] Xu R. (1998). Bioactive peptides in milk and their biological and health implications. Food Rev. Int..

[38] Bilikiewicz A., Gaus W. (2004). Colostrinin (a naturally occurring, proline-rich, polypeptide mixture) in the treatment of Alzheimer’s disease. J. Alzheimer’s Dis..

[39] Kita M., Kobayashi K., Obara K., Koikeda T., Umeda S., Ano Y. (2019). Supplementation with Whey Peptide Rich in beta-Lactolin Improves Cognitive Performance in Healthy Older Adults: A Randomized, Double-Blind, Placebo-Controlled Study. Front. Neurosci..

[40] Wu L., Sun D. (2016). Meta-Analysis of Milk Consumption and the Risk of Cognitive Disorders. Nutrients.

[41] Anderson R.C., Alpass F.M. (2024). Effectiveness of dairy products to protect against cognitive decline in later life: A narrative review. Front. Nutr..

[42] Cheng J., Hu H., Ju Y., Liu J., Wang M., Liu B., Zhang Y. (2024). Gut microbiota-derived short-chain fatty acids and depression: Deep insight into biological mechanisms and potential applications. Gen. Psychiatr..

[43] Guo C., Huo Y.-J., Li Y., Han Y., Zhou D. (2022). Gut-brain axis: Focus on gut metabolites short-chain fatty acids. World J. Clin. Cases.

[44] Motataianu A., Serban G., Andone S. (2023). The Role of Short-Chain Fatty Acids in Microbiota-Gut-Brain Cross-Talk with a Focus on Amyotrophic Lateral Sclerosis: A Systematic Review. Int J. Mol. Sci..

[45] Reimann F., Gribble F.M. (2016). G protein-coupled receptors as new therapeutic targets for type 2 diabetes. Diabetologia.

[46] Dinan T.G., Cryan J.F. (2017). Gut instincts: Microbiota as a key regulator of brain development, ageing and neurodegeneration. J. Physiol..

[47] Socała K., Doboszewska U., Szopa A., Serefko A., Włodarczyk M., Zielińska A., Poleszak E., Fichna J., Wlaź P. (2021). The role of microbiota-gut-brain axis in neuropsychiatric and neurological disorders. Pharmacol. Res..

[48] Silva Y.P., Bernardi A., Frozza R.L. (2020). The Role of Short-Chain Fatty Acids from Gut Microbiota in Gut-Brain Communication. Front. Endocrinol..

[49] Onisiforou A., Charalambous E.G., Zanos P. (2025). Shattering the Amyloid Illusion: The Microbial Enigma of Alzheimer’s Disease Pathogenesis—From Gut Microbiota and Viruses to Brain Biofilms. Microorganisms.

[50] Ansari F., Neshat M., Pourjafar H., Jafari S.M., Samakkhah S.A., Mirzakhani E. (2023). The role of probiotics and prebiotics in modulating of the gut-brain axis. Front Nutr..

[51] Snigdha S., Ha K., Tsai P., Dinan T.G., Bartos J.D., Shahid M. (2022). Probiotics: Potential novel therapeutics for microbiota-gut-brain axis dysfunction across gender and lifespan. Pharmacol. Ther..

[52] Suganya K., Koo B.-S. (2020). Gut–Brain Axis: Role of Gut Microbiota on Neurological Disorders and How Probiotics/Prebiotics Beneficially Modulate Microbial and Immune Pathways to Improve Brain Functions. Int. J. Mol. Sci..

[53] Zheng M., Ye H., Yang X., Shen L., Dang X., Liu X., Gong Y., Wu Q., Wang L., Ge X. (2024). Probiotic Clostridium butyricum ameliorates cognitive impairment in obesity via the microbiota-gut-brain axis. Brain Behav. Immun..

[54] Zhao H., Chen X., Zhang L., Tang C., Meng F., Zhou L., Zhu P., Lu Z., Lu Y. (2023). Ingestion of Lacticaseibacillus Rhamnosus Fmb14 prevents depression-like behavior and brain neural activity via the microbiota–gut–brain axis in colitis mice. Food Funct..

[55] Dicks L.M.T. (2022). Gut Bacteria and Neurotransmitters. Microorganisms.

[56] Bao X., Wu J. (2021). Impact of food-derived bioactive peptides on gut function and health. Food Res. Int..

[57] Martínez-Augustin O., Rivero-Gutiérrez B., Mascaraque C., Sanchez de Medina F.S. (2014). Food derived bioactive peptides and intestinal barrier function. Int. J. Mol. Sci..

[58] Yaffe K., Middleton L.E., Lui L.Y., Spira A.P., Stone K., Racine C., Ensrud K.E., Kramer J.H. (2011). Mild cognitive impairment, dementia, and their subtypes in oldest old women. Arch. Neurol..

[59] Arenaza-Urquijo E.M., Boyle R., Casaletto K., Anstey K.J., Vila-Castelar C., Colverson A., Palpatzis E., Eissman J.M., Ng T.K.S., Raghavan S. (2024). Sex and gender differences in cognitive resilience to aging and Alzheimer’s disease. Alzheimer’s Dement..

[60] Pszczolowska M., Walczak K., Miskow W., Mroziak M., Kozlowski G., Beszlej J.A., Leszek J. (2024). Association between Female Reproductive Factors and Risk of Dementia. J. Clin. Med..

[61] Sattler C., Toro P., Schönknecht P., Schröder J. (2012). Cognitive activity, education and socioeconomic status as preventive factors for mild cognitive impairment and Alzheimer’s disease. Psychiatry Res..

[62] Sharp E.S., Gatz M. (2011). Relationship Between Education and Dementia: An Updated Systematic Review. Alzheimer Dis. Assoc. Disord..

[63] Ashton N.J., Janelidze S., Mattsson-Carlgren N., Binette A.P., Strandberg O., Brum W.S., Karikari T.K., González-Ortiz F., Di Molfetta G., Meda F.J. (2022). Differential roles of Aβ42/40, p-tau231 and p-tau217 for Alzheimer’s trial selection and disease monitoring. Nat. Med..

[64] Devanarayan V., Doherty T., Charil A., Sachdev P., Ye Y., Murali L.K., Llano D.A., Zhou J., Reyderman L., Hampel H. (2024). Plasma pTau217 predicts continuous brain amyloid levels in preclinical and early Alzheimer’s disease. Alzheimer’s Dement..

[65] Palmqvist S., Tideman P., Cullen N., Zetterberg H., Blennow K., Dage J.L., Stomrud E., Janelidze S., Mattsson-Carlgren N., the Alzheimer’s Disease Neuroimaging Initiative (2021). Prediction of future Alzheimer’s disease dementia using plasma phospho-tau combined with other accessible measures. Nat. Med..

[66] Pichet Binette A., Palmqvist S., Bali D., Farrar G., Buckley C.J., Wolk D.A., Zetterberg H., Blennow K., Janelidze S., Hansson O. (2022). Combining plasma phospho-tau and accessible measures to evaluate progression to Alzheimer’s dementia in mild cognitive impairment patients. Alzheimer’s Res. Ther..

[67] Kivipelto M., Solomon A., Ahtiluoto S., Ngandu T., Lehtisalo J., Antikainen R., Bäckman L., Hänninen T., Jula A., Laatikainen T. (2013). The Finnish geriatric intervention study to prevent cognitive impairment and disability (FINGER): Study design and progress. Alzheimer’s Dement..

[68] Stephen R. (2020). The Finnish Geriatric Intervention Study to Prevent Cognitive Impairment and Disability (Finger): Findings from the Structural Brain Mri Sub-Study. Ph.D. Thesis.

