# Supplementary material for: Augmenting Cognitive Function in the Elderly with Mild Cognitive Impairment Using Probiotic Lacticaseibacillus rhamnosus CBT-LR5: A 12-Week Randomized, Double-Blind, Parallel-Group Non-Comparative Study
Source: Nutrients. 2025 Feb 14;17(4):691. doi: 10.3390/nu17040691 (PMC11858765; doi:10.3390/nu17040691)
Supplement: Supplementary file 1 [file nutrients-17-00691-s001.zip › nutrients-3442847-supplementary.pdf]

Supplementary Materials:

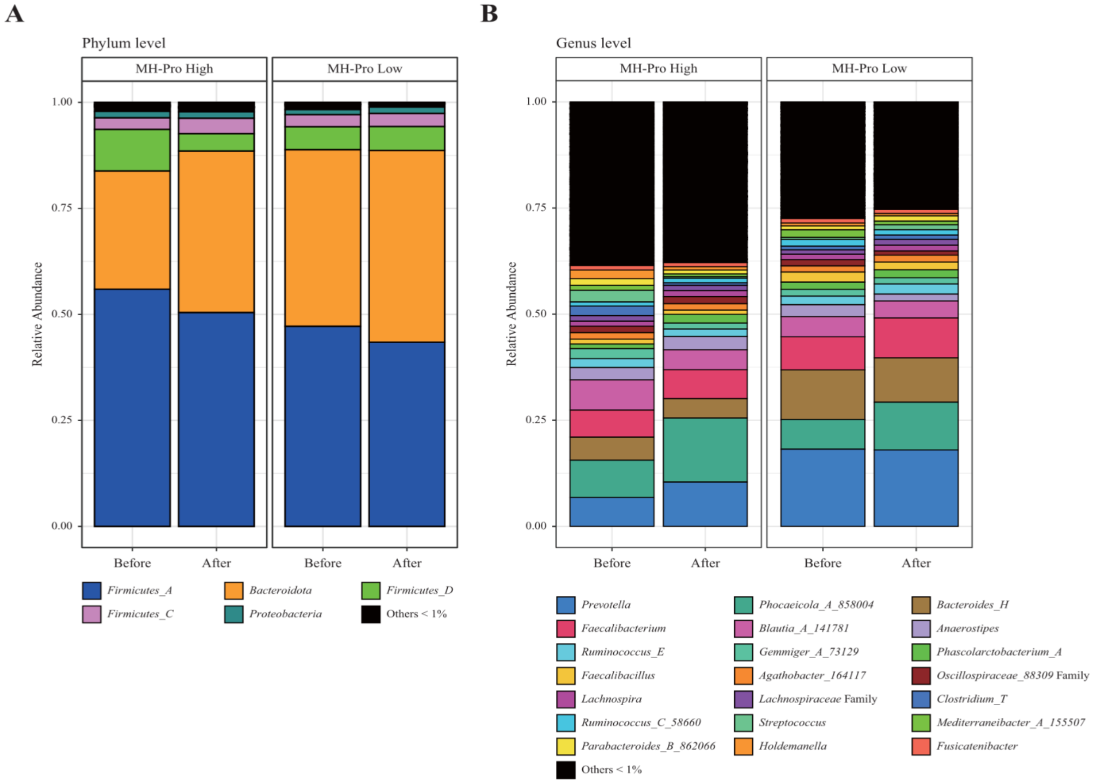

Supplementary Figure S1. Relative abundance of gut microbiota at phylum and genus levels in MH-Pro high and low groups before and after supplementation.

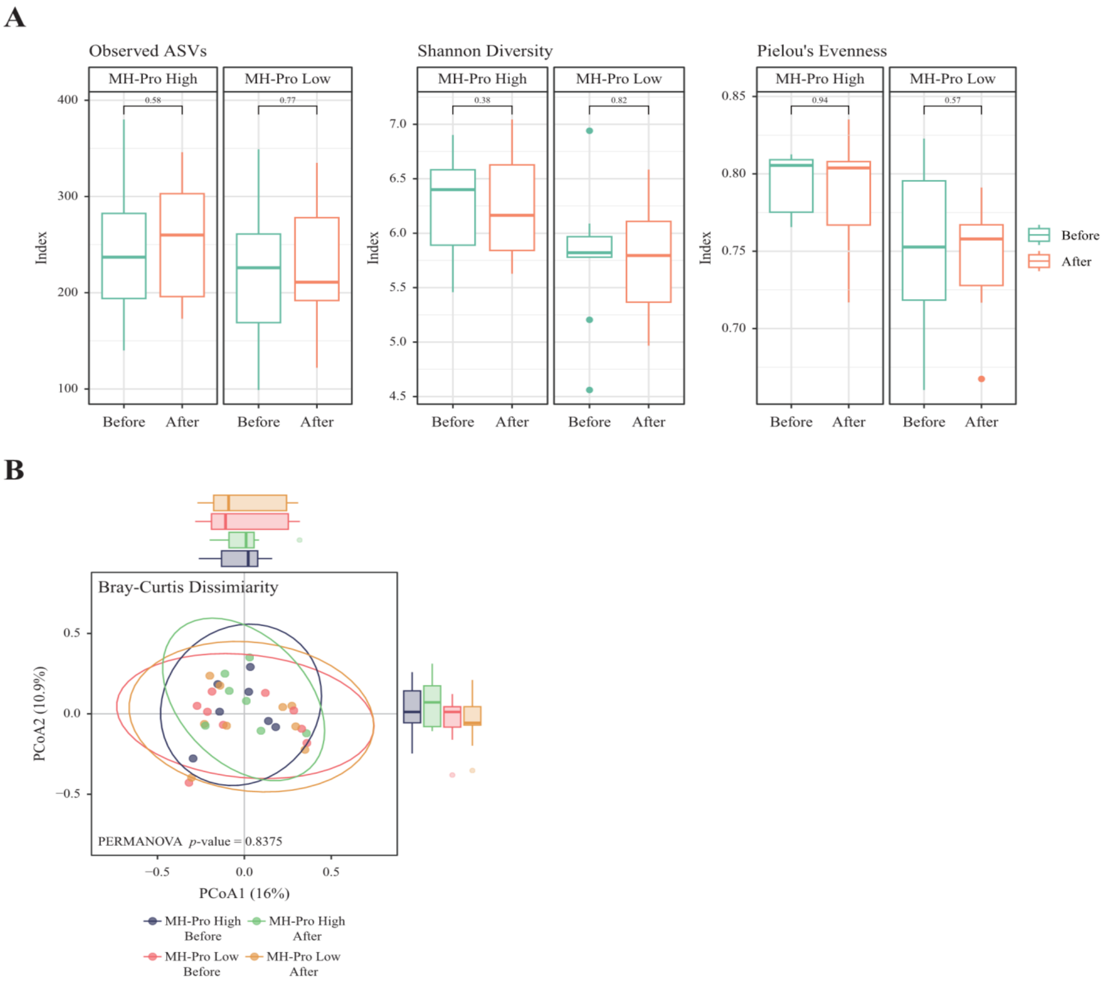

Supplementary Figure S2. Alpha and beta diversity analyses of gut microbiota in MH-Pro high and low groups before and after supplementation.

Supplementary Table S1. Major nutrient intakes and metabolic equivalent values of the intervention groups measured at baseline and 12 weeks.

	MH-Pro high group (n = 9)				MH-Pro low group (n = 9)				
	Baseline	Week 12	Change	*p*-value[1)]	Baseline	Week 12	Change	*p*-value[1)]	*p*-value[2)]
Energy (Kcal)	1,301.20 ± 202.60	1,584.50 ± 271.80	283.30 ± 126.20	0.001	1,689.1 ± 388.80	1,666.40 ± 439.60	-22.70 ± 442.40	0.88	0.08
Carbohydrates (g)	226.10 ± 56.90	255.30 ± 46.90	29.20 ± 35.70	0.040	263.50 ± 95.70	260.70 ± 85.20	-2.80 ± 83.80	0.92	0.32
Lipids (g)	25.80 ± 10.30	39.50 ± 18.40	13.70 ± 12.0	0.009	43.10 ± 20.0	39.50 ± 17.80	-3.70 ± 25.10	0.67	0.08
Protein (g)	48.70 ± 9.40	56.20 ± 11.60	7.60 ± 17.70	0.23	62.90 ± 14.30	64.60 ± 23.0	1.70 ± 23.60	0.83	0.56
Fiber (g)	21.10 ± 10.30	22.40 ± 6.70	1.30 ± 8.80	0.66	24.10 ± 7.80	22.0 ± 4.50	-2.10 ± 7.50	0.43	0.39
MET value (min/week)	1,613.30 ± 920.90	1,577.90 ± 1,187.10	-35.60 ± 624.90	0.87	2,800.0 ± 2,906.50	2,706.70 ± 2,492.60	-93.30 ± 1,049.60	0.80	0.89

Values are presented as mean ± SD. [1)] Analyzed by paired *t* test; [2)] Change values between groups were analyzed by independent t-test; Abbreviation: MET, metabolic equivalent.

Supplementary Table S2. Laboratory profiles of the subjects in this study

Laboratory profiles (standard range)	MH-Pro high group (n = 9)			MH-Pro low group (n = 9)			
	Baseline	Week 12	*p*-value$^{1)}$	Baseline	Week 12	*p*-value$^{1)}$	*p*-value$^{2)}$
WBC (4.8 – 10.8 × 10^3/μL)	4.90 ± 1.10	5.60 ± 2.0	0.27	5.20 ± 1.20	5.10 ± 1.20	0.79	0.29
RBC (4.2 – 5.4 × 100^3/μL)	4.30 ± 0.40	4.40 ± 0.30	0.07	4.20 ± 0.40	4.20 ± 0.40	0.22	0.52
Hemoglobin (12 – 16g/dL)	13.40 ± 1.20	13.60 ± 1.10	0.09	13.20 ± 1.30	13.30 ± 1.20	0.32	0.56
Hematocrit (37 – 47 %)	40.10 ± 3.10	41.70 ± 3.10	0.02	39.90 ± 4.0	41.10 ± 3.50	0.01	0.74
Platelet (130 – 450 × 10^3/μL)	218.50 ± 31.10	220.0 ± 33.70	0.79	226.30 ± 60.80	208.20 ± 52.70	0.06	0.06
ALP (45 – 129 IU/L)	72.40 ± 20.0	71.0 ± 20.10	0.59	72.80 ± 22.90	73.60 ± 28.70	0.87	0.69
GGT (8 – 48 IU/L)	17.30 ± 8.90	20.30 ± 13.0	0.13	20.20 ± 9.20	33.50 ± 45.0	0.40	0.52
AST (12 – 33IU/L)	24.90 ± 4.50	25.80 ± 6.90	0.53	22.0 ± 3.10	24.0 ± 6.20	0.24	0.61
ALT (5 – 35 IU/L)	20.60 ± 9.40	23.30 ± 14.80	0.30	18.0 ± 4.20	19.70 ± 5.70	0.36	0.74
Total bilirubin (0.2 – 1.2 mg/dL)	0.70 ± 0.30	0.70 ± 0.30	0.48	0.60 ± 0.30	0.60 ± 0.30	0.81	0.70
Total protein (6.7 – 8.3 g/dL)	7.10 ± 0.40	7.10 ± 0.40	0.84	7.20 ± 0.30	7.10 ± 0.20	0.22	0.26
Albumin (3.5 – 5.3 g/dL)	4.50 ± 0.30	4.5 ± 0.3	0.31	4.60 ± 0.20	4.50 ± 0.20	0.24	0.12
BUN (8 – 23 mg/dL)	14.50 ± 2.20	15.50 ± 4.10	0.30	13.90 ± 2.90	14.30 ± 3.0	0.71	0.67
Creatinine (0.7 – 1.7 mg/dL)	0.70 ± 0.10	0.70 ± 0.10	0.15	0.70 ± 0.10	0.70 ± 0.10	0.76	0.23
Total cholesterol (~ 200 mg/dL)	182.40 ± 47.10	179.50 ± 44.10	0.42	191.50 ± 28.40	193.70 ± 26.80	0.73	0.48
Triglyceride (~ 150 mg/dL)	92.10 ± 44.70	90.10 ± 31.30	0.81	134.80 ± 40.20	128.50 ± 50.40	0.69	0.81
HDL-cholesterol (~ 40 mg/dL)	65.20 ± 10.40	65.90 ± 12.90	0.78	63.20 ± 14.30	65.0 ± 12.60	0.21	0.69
LDL-cholesterol (~ 100 mg/dL)	111.50 ± 42.90	109.30 ± 38.90	0.33	116.0 ± 25.70	117.90 ± 26.80	0.70	0.75
Glucose (74–106 mg/dL)	90.80 ± 12.40	90.0 ± 16.10	0.76	90.90 ± 8.0	89.10 ± 8.10	0.35	0.75
CK (Male: 190 IU/L	97.0 ± 46.20	106.60 ± 51.10	0.03	136.0 ± 64.60	123.10 ± 59.70	0.14	0.02

Female: 170 IU/L)							
LDH (~ 250 IU/L)	199.40 ± 18.60	202.30 ± 16.0	0.58	225.90 ± 34.40	220.80 ± 26.40	0.55	0.42
Specific gravity (1.005 ~ 1.030)	1.02 ± 0.0	1.02 ± 0.0	0.52	1.02 ± 0.01	1.01 ± 0.0	0.63	0.42
pH (4.5 ~ 9.0)	6.40 ± 0.80	6.40 ± 0.70	0.89	6.20 ± 0.80	6.40 ± 0.80	0.44	0.57

Values are presented as mean ± SD. [1)] Differences between baseline and 12 weeks within each group were analyzed by paired t-test; [2)] Change values between groups were analyzed by independent t-test. Abbreviations: WBC, White Blood Cell; RBC, Red Blood Cell; ALP; Alkaline Phosphatase; GGT, Gamma Glutamyl Transferase; AST, Aspartate Transaminase; ALT, Alanine Transaminase; BUN, Blood Urea Nitrogen; CK, creatine kinase; LDH, lactate dehydrogenase.
